# Identification of transcription factors and single nucleotide polymorphisms of *Lrh1* and its homologous genes in *Lrh1*-knockout pancreas of mice

**DOI:** 10.1186/1471-2350-15-43

**Published:** 2014-04-15

**Authors:** Maochun Tang, Li Cheng, Rongrong Jia, Lei Qiu, Hua Liu, Shu Zhou, Xiuying Ma, Guoyong Hu, Xingpeng Wang, Yan Zhao

**Affiliations:** 1 Department of Gastroenterology, Shanghai Tenth People’s Hospital, Tongji University School of Medicine, No.301, Yanchang Middle Road, Shanghai 200072, China; 2 Department of Gastroenterology, Shanghai First People’s Hospital Affiliated Shanghai Jiaotong University, Shanghai 200080, China

**Keywords:** *Lrh1*-knockout pancreas, RNA-Seq, *Lrh1* homologous gene, Transcription factor, Single nucleotide polymorphisms

## Abstract

**Background:**

To identify transcription factors (TFs) and single nucleotide polymorphisms (SNPs) of *Lrh1* (also named *Nr5a2*) and its homologous genes in *Lrh1*-knockout pancreas of mice.

**Methods:**

The RNA-Seq data GSE34030 were downloaded from Gene Expression Omnibus (GEO) database, including 2 *Lrh1* pancreas knockout samples and 2 wild type samples. All reads were processed through TopHat and Cufflinks package to calculate gene-expression level. Then, the differentially expressed genes (DEGs) were identified via non-parametric algorithm (NOISeq) methods in R package, of which the homology genes of *Lrh1* were identified via BLASTN analysis. Furthermore, the TFs of *Lrh1* and its homologous genes were selected based on TRANSFAC database. Additionally, the SNPs were analyzed via SAM tool to record the locations of mutant sites.

**Results:**

Total 15683 DEGs were identified, of which 23 was *Lrh1* homology genes (3 up-regulated and 20 down-regulated). Fetoprotein TF (FTF) was the only TF of *Lrh1* identified and the promoter-binding factor of FTF was *CYP7A*. The SNP annotations of *Lrh1* homologous genes showed that 92% of the mutation sites were occurred in intron and upstream. Three SNPs of *Lrh1* were located in intron, while 1819 SNPs of *Phkb* were located in intron and 1343 SNPs were located in the upstream region.

**Conclusion:**

FTF combined with CYP7A might play an important role in *Lrh1* regulated pancreas-specific transcriptional network. Furthermore, the SNPs analysis of *Lrh1* and its homology genes provided the candidate mutant sites that might affect the *Lrh1-*related production and secretion of pancreatic fluid.

## Background

The pancreas is an endocrine gland, producing insulin, glucagon, somatostatin, and pancreatic polypeptide, and also an exocrine gland, accounting for more than 98% of pancreatic gland and secreting pancreatic juice containing digestive enzymes [1]. These digestive enzymes help to further break down the carbohydrates, proteins and lipids in the chime and thus support the absorption and digestion of nutrition in small intestine [2]. In the past decades, many research have focused on target genes and transcription factors (TFs) involved in the exocrine pancreas-specific transcriptional networks which are required for the production and secretion of pancreatic fluid that helps out the digestive system. Currently, many exocrine pancreas-specific genes and transcription factors have been identified, which may promote the understanding of the effect of exocrine pancreas on digestive system.

Liver receptor homolog-1 (Lrh1; also called Nr5a2) is a nuclear receptor of ligand-activated transcription factors in liver by binding as a monomer to DNA sequence elements with the consensus sequence 5′-Py-CAAGGPyCPu-3′ [3]. It has been suggested that *Lrh1* is progressively expressed in both the endocrine and exocrine pancreas [4]. Baquié M et al. [5] have found that *Lrh1* is expressed in human islets and protects β-cells against stress-induced apoptosis that may be mediated via the increased glucocorticoid production that blunts the pro-inflammatory response of islets. Meanwhile, Fayard E et al. [6] have demonstrated that both *Lrh1* and *CEL* (encoding carboxyl ester lipase) are co-expressed and confined to the exocrine pancreas. The identification of *CEL* as an *Lrh1*-target gene indicates that *Lrh1* plays an important role in enterohepatic cholesterol homeostasis associated with the absorption of cholesteryl esters and the assembly of lipoproteins by the intestine [7]. Besides, *Lrh1* is a downstream target in the *PDX-1* (lead to pancreas agenesis) regulatory cascade that is activated only during early stages of pancreas development and that governs pancreatic development, differentiation and function [8].

Recently, the rapid advent of next-generation sequencing has made this technology broadly available for researchers in various molecular and cellular biological fields. Holmstrom SR et al. [9] have determined the cistrome and transcriptome for the nuclear receptor LRH-1 in exocrine pancreas and revealed that *Lrh1* directly induces expression of genes encoding digestive enzymes and secretory and mitochondrial proteins based on Chromatin immunoprecipitation (ChIP)-seq and RNA-seq analyses. Besides, *Lrh1* cooperates with the pancreas transcription factor 1-L complex (PTF1-L) in regulation of exocrine pancreas-specific gene expression. However, many potential target genes and TFs of *Lrh1* based on RNA-seq analysis have not been revealed.

In the present study, we downloaded the raw RNA-seq data of Holmstrom SR et al. deposited in The National Center for Biotechnology Information (NCBI) database, which were analyzed using multiple bioinformatics tools in the purpose of finding specific TFs of *Lrh1* and its homology genes. Additionally, we also annotated the SNPs of *Lrh1* and its homology genes to predict their mutant sites. Our study might improve the understanding of the regulation network of *Lrh1*-related production and secretion of pancreatic fluid.

## Methods

### RNA-seq data acquisition

The RNA-seq data was downloaded from NCBI (http://www.ncbi.nlm.nih.gov/) Gene Expression Omnibus (GEO) database (GEO accession: GSE34030 [9]), including 2 *Lrh1* pancreas knockout samples and 2 wild type samples. RNA preparations were subjected to the Illumina RNA-seq protocol and the platform was GPL9185.

### Data pre-processing, gene expression and homology gene of *Lrh1*

The raw data were downloaded from SRA (Sequence Read Archive) of NCBI and then converted to fastq reads using fastq-dump program of NCBI SRA Toolkit (−q 64) (http://trace.ncbi.nlm.nih.gov/Traces/sra/sra.cgi?view=std). Then, these reads were processed through TopHat [10] and Cufflinks [11] package to calculate gene-expression level. All parameters were set up according to the default settings of TopHat and Cufflinks. The DEGs were identified via non-parametric algorithm (NOISeq) methods in R package [12]. The thresholds value was False Discovery Rate (FDR) < 0.001. BLASTN analysis [13,14] of the selected DEGs was used to identify the homology genes of *Lrh1*. Homology genes here refer to the paralogous genes which share a high degree of sequence similarity (maximum expectation value was set to e^−5^) with *Lrh1* in mice.

### Function annotation of *Lrh1* homologous genes

For functional analysis of *Lrh1* homologous genes, DAVID (Database for Annotation, Visualization and Integrated Discovery) [15] was performed for Gene Ontology (GO) [16] function and Kyoto Encyclopedia of Genes and Genomes (KEGG) pathway enrichment analysis.

### Transcription factor (TF) of *Lrh1* homologous genes

Combined with TRANSFAC database [17], the TFs regulated the transcription of *Lrh1* and its homologous genes were identified. Then, the promoter-binding factors regulated via the selected TFs were analyzed based on the website (http://www.nursa.org/molecule.cfm?molType=receptor&molId=5A2).

### Screening of SNPs

The fastq reads were mapped to marker sequences using bowtie [18]. And the aligned reads were called using the SAM tool [19]. In order to minimize the risk of false-positive SNP Callings, the threshold value was that ID was “*” with quality > 50, or ID was not “*” with quality > 20. These SNPs were annotated via SnpEff [20] to categorize the effects of variants in genome sequences. The identified SNPs were searched in the dbSNP database to identify diseased SNPs or de novo discovered SNPs.

## Results

### Identification and homology analysis of differentially expressed genes

After data processing, at FDR < 0.001, a total of 15683 DEGs were identified, including 10994 up-regulated and 4698 down-regulated genes. BLASTN analysis of DEGs showed 23 *Lrh1* homology genes. Among them, 3 were up-regulated and 20 were down-regulated (Table 1).

**Table 1:** ***Lrh1 *** **homology genes of differentially expressed genes**

**Regulation**	***Lrh1 *** **homology genes**
Up-regulated	Fzd5, Klrb1f, Phc1
Down-regulated	March8, Zfp282, Atrx, Sos2, Zfc3h1, Pofut1, Gm9079, Ykt6, Phkb, Galc, Setd1a, Fzd5, Kazn, Kcnmb1, Lamc2, Mylk4, Ache, Pbxip1, Lrh1, Phc1

### Function and pathway annotation of *Lrh1* homologous genes

**Figure 1:**
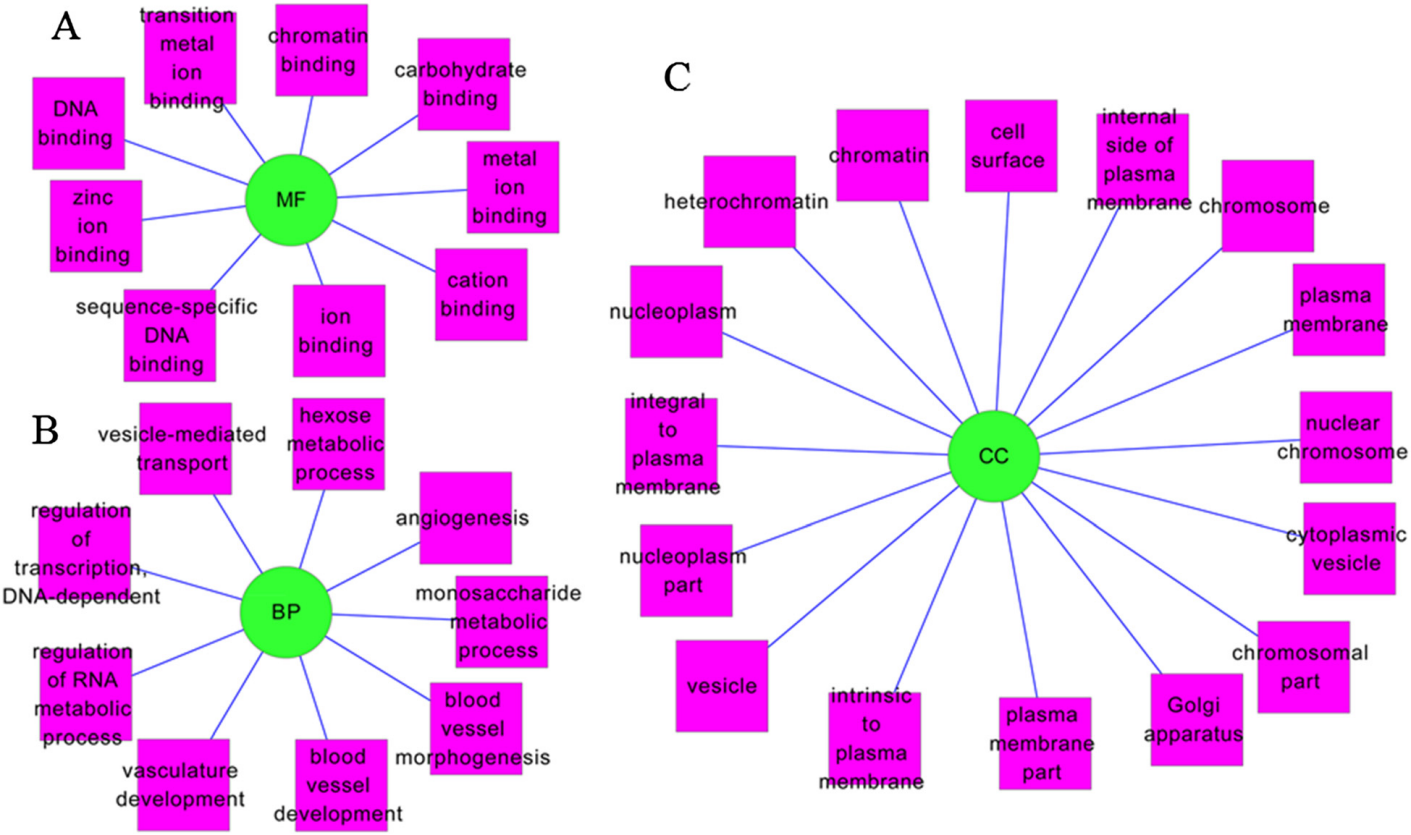
**GO functional annotation of *Lrh1 *and its homology genes.** GO terms included Molecular Function (MF) GO-terms **(A)**, Biological Process (BP) GO-terms **(B)** and Cellular Component (CC) GO-terms **(C)**.

**Figure 2:**
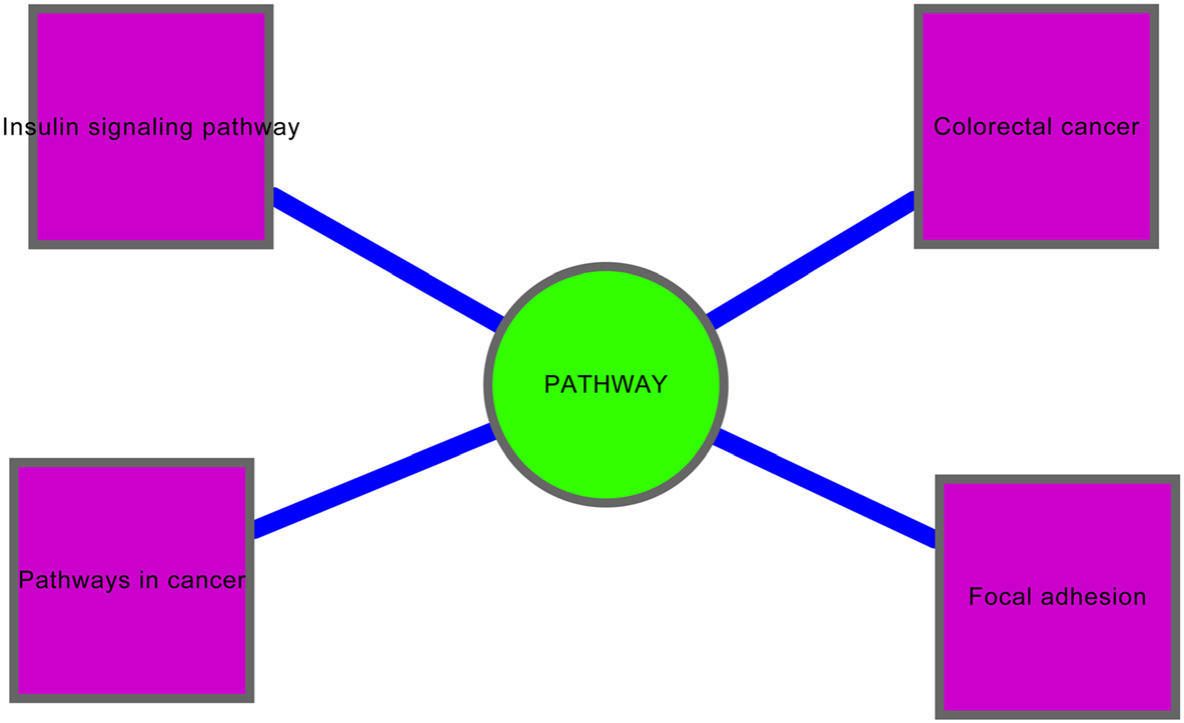
**KEGG pathway annotations of *Lrh1 *** **and its homology genes.**

To determine the function of *Lrh1* homologous genes in pancreas, GO enrichment analysis and KEGG pathway enrichment analysis were used to analyze the up- and down-regulated Lrh1 homologous genes. For function and pathway annotation, DEGs were enriched into hexose metabolic process and monosaccharide metabolic process, which were involved into glycometabolism (Figure 1). Meanwhile, KEGG pathway enrichment analysis identified insulin signaling pathway, indicating that the disorders of glycometabolism might be resulted from insulin resistance and/or insulin secretion (Figure 2). *PHKB*, an *Lrh1* homologous gene, participated in GO terms (hexose metabolic process and monosaccharide metabolic process) and KEGG pathway (insulin signaling pathway), was identified.

### Potential TFs of *Lrh1* homologous genes

Fetoprotein transcription factor (FTF) (ID: T04754) of *Lrh1* was the only TF identified based on TRANSFAC database. Meanwhile, the promoter-binding factor of *Lrh1* was *CYP7A* (Cholesterol 7α-hydroxylase).

### SNPs of *Lrh1* homologous genes

The annotation of SNPs of *Lrh1* homologous genes showed that the majority of SNPs were located in intron and upstream, accounting for nearly 92% of all SNPs (Tables 2 and 3). Three SNPs of *Lrh1* were distributed in intron. Meanwhile, total 1819 SNPs of *Phkb* were located in the intron and 1343 SNPs were located in the upstream region of *Phkb*.

**Table 2:** **The number of different type of SNPs of *Lrh1 *** **and its homology genes**

**Type**	**Number**	**Proportion**
Downstream	581	0.052234
Intron	8251	0.741796
Non-synonymous-coding	122	0.010968
Splice-site-donor	2	0.00018
Start-gained	4	0.00036
Stop-gained	5	0.00045
Synonymous-coding	97	0.008721
Upstream	1975	0.17756
UTR-3-prime	79	0.007102
UTR-5-prime	7	0.000629

**Table 3:** **The annotation of SNPs of *Lrh1 *** **and its homology genes**

**Gene name**	**Chromo**	**Number**	**Position**
Zfc3h1	10	4	115385510
Galc	12	36	98232394-98232429
Mylk4	13	13	32711300-32711312
Fzd5	1	22	64737712-64737722
Lrh1	1	6	136849713-136870578
Lamc2	1	923	153145967-153191429
Pofut1	2	188	153239719-153275253
Ache	5	19	137288432-137288449
Zfp282	6	1016	47876551-47913485
8-Mar	6	4231	116342541-116360897
Setd1a	7	1834	127778726-127802950
Phkb	8	3197	85837045-86008353
Atrx	X	7	105863283-105906922

## Discussion

In the present study, combined with RNA-seq data of *Lrh1*-knockout pancreas samples, FTF was the only TF of *Lrh1* identified based on TRANSFAC database and may regulate cholesterol catabolism into bile acids by activation of the promoter-binding factor *CYP7A*. Many literatures have elucidated the function of *Lrh1*/*Nr5a2*/*FTF*/*CYP7A* via experimental studies [21-25].

*FTF* is highly expressed in the liver and intestine and is implicated in the regulation of cholesterol, bile acid and steroid hormone homeostasis [26]. Nearly 50% of the body cholesterol is catabolized to bile acids via bile acid biosynthetic pathway, of which cholic acid (hydroxylated at position 12) and chenodeoxycholic acid are the major primary bile acids and play an important role in cholesterol homeostasis [19]. Chenodeoxycholic acid can repress *FTF* expression and is a more potent suppressor of HMG-CoA reductase and cholesterol 7α-hydroxylase/CYP7A1 (7α-hydroxylase) than cholic acid [27]. It has been proposed that *Lrh1*, also known as CYP7A promoter-binding factor, *LRH1*, or *FTF*, is required for the transcription of the 7α-hydroxylase gene [19,28]. The small heterodimer partner 1 (SHP) of the nuclear bile acid receptor, FXR (farnesoid X receptor) can dimerize with FTF and diminish its activity on the 7α-hydroxylase promoter [29].

Although *Lrh1* has been demonstrated the function in feedback regulation of *CYP7A1* expression as part of the FXR-SHP-LRH-1 cascade, in which bile acids can inhibit their own synthesis, the mechanisms have not been well understood. Out C et al. [25] have suggested that *CYP7A1* expression is increased rather than decreased under chow-fed conditions in *Lrh1*-knockdown mice that is coincided with a significant reduction in expression of intestinal Fgf15, a suppressor of *CYP7A1*. Besides, Noshiro M et al. [30] have suggested that the circadian rhythm of *CYP7A* is regulated by multiple transcription factors, including DBP, REV-ERBα/β, LXRα, HNF4α DEC2, E4BP4, and PPARα. Hepatocyte nuclear factor 4α (HNF4α) and FTF are two major TFs driving *CYP7A1* promoter activity in lipid homeostasis. Bochkis IM et al. [31] have shown that prospero-related homeobox (Prox1) directly interacts with both HNF4α and FTF and potently co-represses *CYP7A1* transcription.

In the present study, we annotated the SNPs of *Lrh1* and its homologous genes, showing that the majority was located in intron and upstream. Quiles Romagosa MÁ [32] has reported that a functional SNP located in *Lrh1* promoter is related to Body Mass Index (BMI) and these SNPs might play important roles in the obese phenotype. However, previous researches mostly focused on SNPs associated with pancreatic cancer cell growth and proliferation. For example, a previous genome-wide association study has identified five SNPs on 1q32.1 associated with pancreatic cancer that mapped to *Lrh1* gene and its up-stream regulatory region [33].

## Conclusions

In conclusion, FTF combined with *CYP7A* might play an important role in *Lrh1* regulated pancreas-specific transcriptional network. Furthermore, the SNPs analysis of *Lrh1* and its homology genes provided the candidate mutant sites that might affect the *Lrh1*-related production and secretion of pancreatic fluid. These common susceptibility loci for *Lrh1* and its homologous genes needed follow-up studies.

### Highlights

1. Total 15683 DEGs were identified, of which 23 was *Lrh1* homology genes (3 up-regulated and 20 down-regulated).

2. Fetoprotein TF was the only TF of *Lrh1* identified based on TRANSFAC database and the promoter-binding factor of fetoprotein TF was CYP7A.

3. The SNP annotations of *Lrh1* homologous genes showed that 92% of mutation sites were occurred in intron and upstream. Three SNPs of *Lrh1* were located in intron, while 1819 SNPs of *Phkb* were located in intron and 1343 SNPs were located in upstream region.

## Competing interest

The authors declare that they have no competing interests.

## Authors’ contributions

MT, XM and CL participated in the design of this study, and they both performed the statistical analysis. RJ, GYH and YZ carried out the study, together with LQ, collected important background information, and drafted the manuscript. HL, XPW and ZS conceived of this study, and participated in the design and helped to draft the manuscript. All authors read and approved the final manuscript.

## Pre-publication history

The pre-publication history for this paper can be accessed here:

http://www.biomedcentral.com/1471-2350/15/43/prepub
